# Time Trends and Factors Associated with Antibiotic Prescribing in Swiss Primary Care (2008 to 2020)

**DOI:** 10.3390/antibiotics9110837

**Published:** 2020-11-23

**Authors:** Nahara Anani Martínez-González, Stefania Di Gangi, Giuseppe Pichierri, Stefan Neuner-Jehle, Oliver Senn, Andreas Plate

**Affiliations:** 1 Institute of Primary Care, University of Zurich and University Hospital of Zurich, Pestalozzistrasse 24, CH-8091 Zurich, Switzerland; stefania.digangi@usz.ch (S.D.G.); giuseppe.pichierri@usz.ch (G.P.); stefan.neuner-jehle@usz.ch (S.N.-J.); oliver.senn@usz.ch (O.S.); andreas.plate@usz.ch (A.P.); 2 Department of Health Sciences and Medicine, University of Lucerne, Frohburgstrasse 3, PO Box 4466, CH-6002 Lucerne, Switzerland

**Keywords:** antibiotic prescriptions, antibiotic prescribing, antibiotic use, primary care, general practice, family medicine, outpatient care, ambulatory care, Switzerland

## Abstract

Antibiotic resistance (ABR) is a major threat to public health, and the majority of antibiotics are prescribed in the outpatient setting, especially in primary care. Monitoring antibiotic consumption is one key measure in containing ABR, but Swiss national surveillance data are limited. We conducted a retrospective cross-sectional study to characterise the patterns of antibiotic prescriptions, assess the time trends, and identify the factors associated with antibiotic prescribing in Swiss primary care. Using electronic medical records data, we analysed 206,599 antibiotic prescriptions from 112,378 patients. Based on 27,829 patient records, respiratory (52.1%), urinary (27.9%), and skin (4.8%) infections were the commonest clinical indications for antibiotic prescribing. The most frequently prescribed antibiotics were broad-spectrum penicillins (BSP) (36.5%), fluoroquinolones (16.4%), and macrolides/lincosamides (13.8%). Based on the WHO AWaRe classification, antibiotics were 57.9% Core-Access and 41.7% Watch, 69% of which were fluoroquinolones and macrolides. Between 2008 and 2020, fluoroquinolones and macrolides/lincosamides prescriptions significantly declined by 53% and 51%; BSP prescriptions significantly increased by 54%. Increasing patients’ age, volume, and employment level were significantly associated with antibiotic prescribing. Our results may inform future antibiotic stewardship interventions to improve antibiotic prescribing.

## 1. Introduction

Antibiotic resistance (ABR) and its consequences have escalated into a serious threat to public health globally [1,2]. A key modifiable driver of the accelerated spread of ABR is the irrational consumption of antibiotics in general, and the (excessive) use of broad-spectrum antibiotics in particular [3,4,5]. While the largest volume of antibiotics used in humans is prescribed in outpatient care, 80–90% of such prescriptions are issued by general practitioners (GP), and up to 50% of these prescriptions are inappropriate [3,5,6]. This excessive antibiotic prescribing comprises a large amount of overall antibiotic use, and it is a major challenge to healthcare systems [7]. Despite government calls and implementation of guidelines, both the global patterns of antibiotic prescribing and the rates of antibiotic consumption in high-income countries have suffered little change over the past years [7,8,9].

One of the top five priorities of the European Center for Disease Prevention and Control (ECDC) and the WHO global action plan to optimise the use of antimicrobials includes gathering data on antibiotic resistance and consumption to improve surveillance as a central element in containing ABR [8,10,11]. Several strategies matching these objectives have established the platforms to monitor resistance and consumption [8,12,13]. Generating knowledge about these patterns can help in determining the appropriateness of antibiotic use. At the local and national levels, this knowledge is crucial to inform the design, implementation and monitoring of antibiotic stewardship, the goal of which is to optimise patient safety and outcomes [14]. As primary care continues to exceed the rates of antibiotic use in hospital care, addressing knowledge gaps about the extent of antibiotic consumption in primary care is of an utmost priority.

Antibiotic consumption in the outpatient care setting in Switzerland is lower than in many of the European countries collaborating in the project of the European Surveillance of Antibiotic Consumption (ESAC) by the ECDC [15,16,17]. It is unclear, however, how much of the prescribing corresponds to primary care only. At the national level, there is no database that allows a systematic collection of data to monitor disease-specific antibiotic consumption in primary care—as yet.

In Switzerland, the Federal Office of Public Health (FOPH) and the Swiss Centre for Antibiotic Resistance (ANRESIS) collect antibiotic consumption data both from the inpatient and outpatient settings [15]. The Swiss surveillance for the outpatient setting feeds from datasets of pharmacies and self-dispensing physicians, or only pharmacies [15]. The outpatient setting, however, comprises services provided on an ambulatory basis including, but not limited to, primary and specialised care. Currently, national surveillance data do not distinguish between these settings. Swiss primary care encompasses about 45% of the outpatient care sector [18]. Research shows that there can be substantial differences in the rates of antibiotic prescribing between outpatient care services, e.g., primary care, out-of-hours care or specialised care [19,20]. Further categorisation by outpatient services in the Swiss surveillance data would be ideal for assessing these differences properly.

Primary research on the study of antibiotic consumption in Swiss primary care is also limited. A few observational studies have provided some insights into the prevalence, predictors or quality of antibiotic prescribing in Swiss primary care, but these focused on specific clinical indications or a specific type (e.g., low or high) of prescribers [21,22,23,24,25].

We aimed to characterise the patterns of antibiotic prescriptions, assess the time trends of these patterns, and identify the factors associated with antibiotic prescribing in Swiss general practice using electronic medical records (EMR) fed by a network of GPs. The EMRs are part of a database project for research in family medicine currently running in German-speaking areas of Switzerland and has so far aggregated patient health routine data over 12 years [26].

## 2. Results

**Figure 1:**
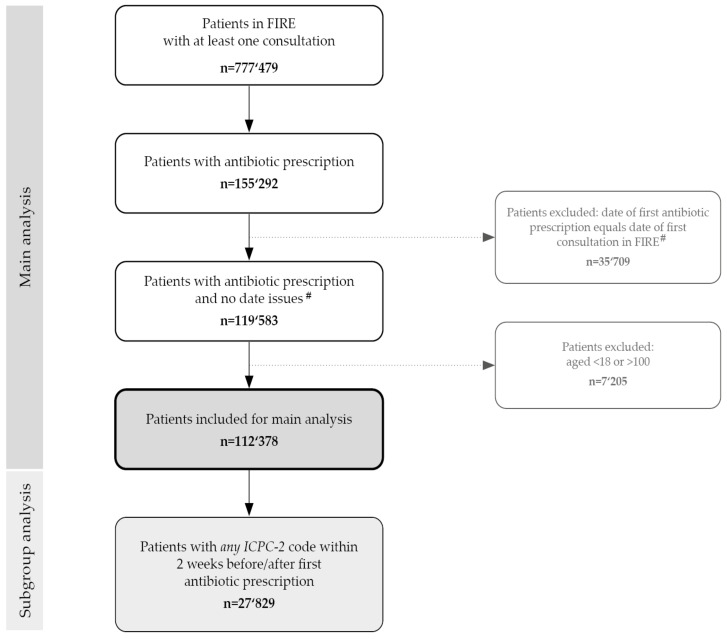
Event flow chart. Number of patients with antibiotic prescriptions in Swiss primary care from the FIRE database. FIRE, Family Medicine ICPC-Research using EMR; EMR, Electronic Medical Records; ICPC, International Classification of Primary Care (-2) code system. #Date issues: when the date of a first antibiotic prescription coincided with the date of a first consultation; a prescription could have been carried over from a previous consultation prior to the patient’s inclusion in the database.

We identified a total of 155,292 patients with antibiotic prescriptions (Figure 1). Eligible data for our analyses comprised 112,378 individual patients with 206,599 prescriptions of systemic antibiotic. The patients’ mean age was 52.9 (SD 20.5; range: 18–100) years, and 60% (n = 67,439) were women. The overall mean number of antibiotic prescriptions per patient was 1.65 (range: 1–28, SD 1.28). Overall, regardless of clinical indications, more antibiotics were prescribed in women compared to men (mean no.: 1.71 (SD 1.29) vs. 1.57 (SD 1.07; *p* < 0.001).

Prescribing data by the canton of practicing GPs were available for 96.0% (198,272) of the prescriptions. Of these, 99.8% (n = 197,949) of the prescriptions were issued in the German-speaking areas, including cantons of different languages but where German is officially the dominant language. The other 0.2% (n = 323) of the prescriptions were issued in cantons where French is officially the dominant language.

### 2.1. Patterns of Antibiotic Prescriptions

Broad-spectrum penicillins (BSP) were the most common class of antibiotics used, accounting for 36.5% (n = 75,534) of the prescriptions among all classes (Table 1). Amoxicillin plus beta-lactamase inhibitors (BLI) were the most frequently prescribed antibiotics both within this class and overall accounting for 29.8% (n = 61,733) of all prescriptions (see Supplementary Materials). Fluoroquinolones and macrolides/lincosamides were the second and third most common classes of antibiotics accounting for 16.4% (n = 33,900) and 13.8% (n = 28,589) of the prescriptions, respectively. The most frequently prescribed antibiotics within these classes were ciprofloxacin and clarithromycin, accounting for 10.5% (n = 21,770) and 7.1% (n = 14,633) of the antibiotic prescriptions, respectively. Based on the WHO Aware classification, 57.9% (n = 119,649) of all prescriptions were “Access” antibiotics, 41.7% (n = 86,142) were “Watch” antibiotics, and 0.02% (n = 48) were “Reserve” antibiotics.

**Figure 2:**
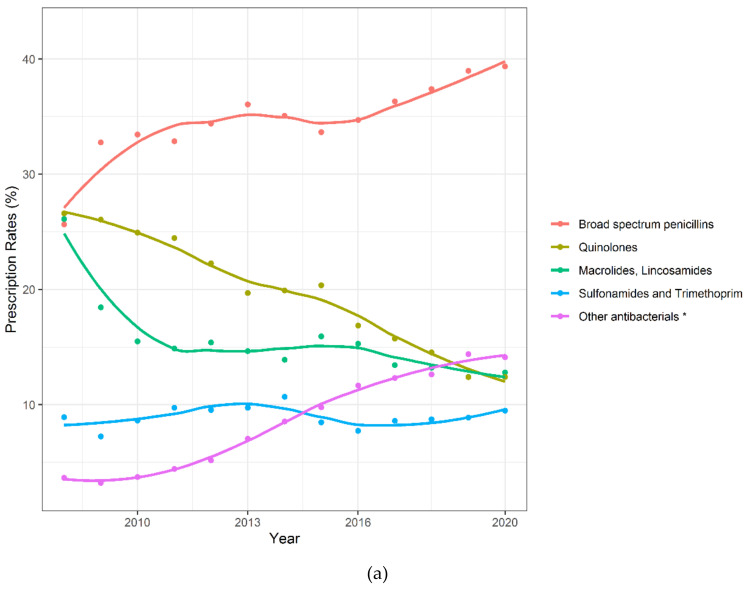

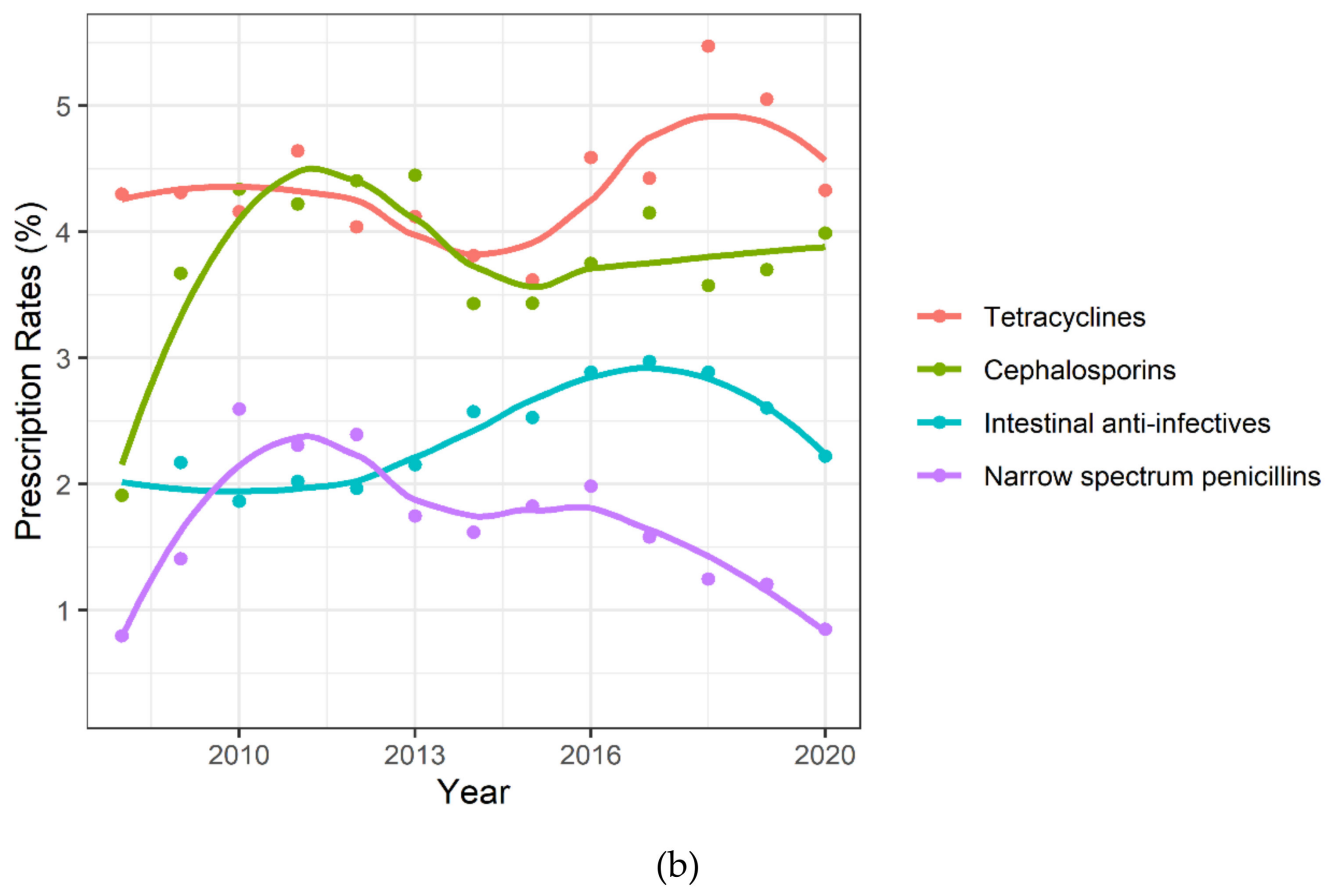
Time trends in antibiotic prescribing in primary care. Points are yearly observed numbers and lines are smoothed curves. (**a**) Antibiotics with >10,000 prescriptions; (**b**) Antibiotics with >1000 prescriptions. * This group contains prescriptions from the ATC group J01XB-J01XX. The prescriptions reported in this group consist of >98% nitrofurantoin and fosfomycin prescriptions.

The distribution of antibiotic prescribing significantly changed over twelve years, from 2008 to 2020 (Figure 2a). Prescriptions of BSP and “other antibacterials” (WHO Anatomical Therapeutic Chemical Classification System (ATC) class J01XB-J01XX: >98% are nitrofurantoin and fosfomycin) significantly increased by 54% (39.3% to 25.6%; yearly linear increase: 0.76 points; *p* < 0.001) and almost tripled (3.7% to 14.1%; linear yearly increase: 1.06 points; *p* < 0.001). At the same time, there was a constant decline in the rates of fluoroquinolone and macrolide/lincosamide. Prescriptions of such antibiotics significantly reduced by 53% (26.6% to 12.4%; yearly linear decrease: −1.27 points, *p* < 0.001) and 51% (26.1% to 12.8%; yearly linear decrease: −0.67 points, *p* = 0.004) respectively. Tetracyclines show a significant linear increase of 0.07 (*p* = 0.006) while cephalosporins, intestinal antiinfectives and narrow-spectrum penicillins showed a wide variation but no significant difference in the rates of prescribing (*p* = 0.182, *p* = 0.458 and *p* = 0.254) over the twelve years (Figure 2b).

**Figure 3:**
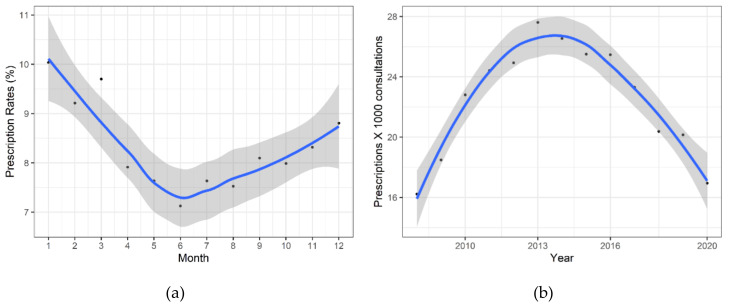
Distribution of antibiotic prescribing in Swiss primary care. Points are observed numbers and lines are smoothed curves; 95% Confidence Interval band is shown in grey. (**a**) Seasonal variation of antibiotic prescriptions by month: 1 = January, 12 = December; (**b**) Fluctuation of antibiotic prescriptions over 12 years.

Distribution of antibiotic prescribing showed a significant variation in the number of prescriptions by month (*p* < 0.001) (Figure 3a). There was a non-monotonic trend in the number of prescriptions throughout the year with the summer months showing the lowest rates of antibiotic prescriptions (23.4%, n = 48,417 for the season). The number of prescriptions increased the most during the cold season (September to December), reaching the highest rates in winter (28.1%, n = 58,060 for the season).

Fluctuation of antibiotic prescribing by year showed that the year 2013 was the observed peak of antibiotic prescriptions (Figure 3b). From 2008 to 2013, there was a steady, significant quadratic increase of 70% (yearly quadratic trend: 16.2 to 27.6; *p* = 0.008) in the number of antibiotic prescriptions per 1000 consultations. From 2013 to 2020, the rate significantly fell by 39% (yearly quadratic trend: 27.6% to 16.95%; *p* = 0.008) prescriptions per 1000 consultations.

### 2.2. Subgroup Analysis

A subgroup of 27,829 (24.7%) patient records contained information by the International Classification of Primary Care 2 (ICPC-2) code system allowing the identification of fifteen clinical indications for which antibiotics were most prescribed. Based on ICPC-2 codes for both general (Table 2) and infectious (Table 3) diseases, respiratory tract infections/symptoms were the most common clinical indication accounting for 52.1% (n = 11,039) of all antibiotics prescribed among the fifteen indications, followed by urinary tract infections and skin infections with 27.9% (n = 5898) and 4.8% (n = 1000) of the antibiotics prescribed respectively.

**Figure 4:**
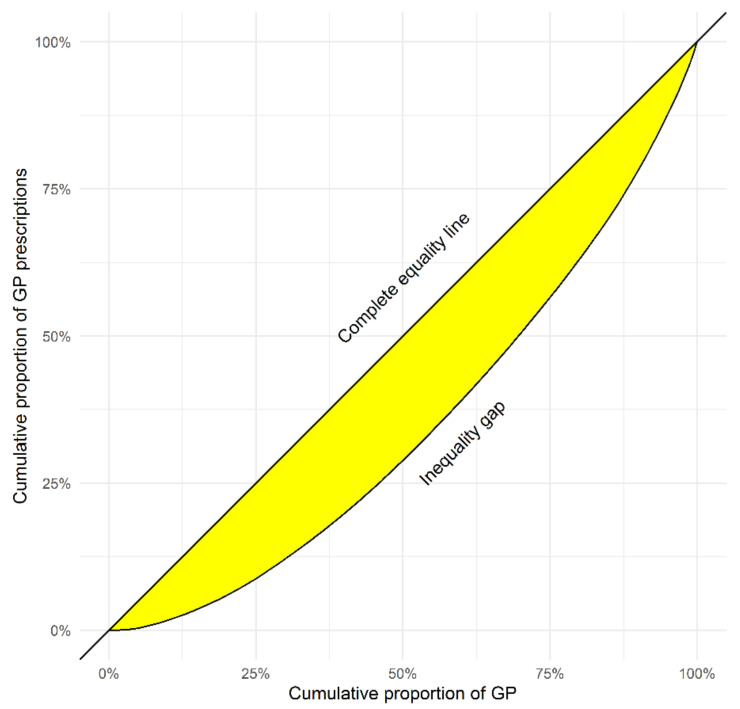
Inequality in prescriptions across Swiss general practitioners: Lorenz curve of antibiotic prescriptions in a subgroup of 240 general practitioners (GPs) with 26,595 antibiotic prescriptions issued in 2018. Index of inequality by Gini’s coefficient = 0.31; “0” = perfect equality (straight line) and “1” = total inequality. The further the Lorenz curve deviates from perfect equality, the higher the Gini coefficient and the inequality.

The index of inequality in the rate of prescriptions across 240 GPs with 26,595 prescriptions is presented in Figure 4. Quantitative differences among GPs show that 25% of the GPs with the lowest antibiotic prescribing accounted for 9% (n = 2303) of all prescriptions—the lower quarter. The 25% of the GPs with the highest prescribing accounted for 43% (n = 11,436) of all prescribed antibiotics—the upper quarter.

### 2.3. Multivariable and Sensitivity Analyses

Regression analysis demonstrated a significant correlation between several factors and antibiotic prescribing in the multivariable analysis (Table 4). At the GP level, antibiotic prescribing was significantly and directly associated with an increasing number of GP consultations (0.281, 95% confidence intervals (CI) 0.163 to 0.398; *p* < 0.001), i.e., as the number of consultations increased, the number of prescriptions also increased. Similarly, GPs employed at a level of <50% prescribed significantly fewer antibiotics (−0.279, 95%CI −0.507 to −0.051; *p* = 0.017) compared to GPs employed full-time. At the patient level, increasing patient age (units of increase or age: 0.002, 95% CI 0.001 to 0.003; *p* < 0.001) and being female (male gender: −0.234, 95%CI −0.272 to −0.196; *p* < 0.001) were significantly associated with more antibiotic prescriptions. Sensitivity analysis showed, however, that after excluding the sample of patients with ICPC codes available for UTIs, gender was no longer associated with antibiotic prescribing (male gender: −0.0281 95%CI −0.111 to 0.0546, *p* = 0.505). Thus, in our analysis, UTIs were a confounding variable for antibiotic prescribing in women.

## 3. Discussion

To the best of our knowledge, our study is the first assessing both the patterns of antibiotic prescribing and its associated factors exclusively in Swiss primary care with a network of GPs as the source of health data. As such, our study contributes by adding data specific to the GP setting, which in national reports is not distinguished from outpatient care [15]. Our study represents a network of GPs working predominantly in the German-speaking areas of Switzerland and a population of adults, the majority of whom were middle-aged and mostly women.

Regardless of the clinical indication, women were more likely to receive antibiotics than men. The three most common clinical indications for antibiotics were, in descending order of frequency, RTIs, UTIs and skin infections. The most frequently prescribed antibiotics were amoxicillin plus BLI, ciprofloxacin and clarithromycin. We found a constant and significant increase in prescriptions of BSP and a significant and steady decline in quinolones prescriptions.

Monitoring antibiotic consumption in both the in- and out-patient settings is of utmost importance as it provides the basis for the implementation and evaluation of antibiotic stewardship interventions. Antibiotic consumption, especially the inappropriate use of antibiotics, is associated with increased rates of ABR [3]. In Europe, nearly 672,000 infections with resistant bacteria were reported in 2015, emphasising the burden of ABR [1]. In the outpatient setting, the distinction between primary and specialised care is critical since prescribing patterns differ significantly, and the majority of antibiotics is prescribed in primary care [19].

Our findings on the most common antibiotic classes used in Swiss primary care are in line with both national and European surveillance programs [15,27]. BSP, fluoroquinolones, macrolides, lincosamides, and tetracyclines are the antibiotic classes most commonly used in the community. The relative order in which these are used might differ across countries. In particular, our study with a focus on primary care shows a distribution of antibiotic use similar to that from the national surveillance, which is based on several outpatient care services [15].

The WHO recently introduced the AWaRe classification to emphasise the importance of the optimal use of antibiotics and their potential for ABR [28]. It recommends using the group of Core-Access antibiotics in a proportion of more than 60% to reduce ABR while achieving a positive impact on health outcomes. In our study, 58% of all prescribed antibiotics classified as Core-Access, which is lower than the proportion (65%) reported in 2018 using Swiss surveillance outpatient health data [15]. The 42% of Watch antibiotics, of which 69% were fluoroquinolones and macrolides, surpasses the 25% reported in 2018. The federal health dataset from the outpatient care setting (specialist and primary care) comprises pharmaceutical sales data and data from pharmacies and self-dispensing physicians. In this dataset, antibiotic prescriptions in primary care are not distinguished from the whole outpatient care setting. Additionally, as the AWaRe classification has evolved, a group of antibiotics initially categorised as “others” (10%) in the 2018 national report are now grouped into specific categories, which affects their final distribution.

Our study shows a steady decline in the rate of antibiotic prescriptions per 1000 patients, which is consistent with the trends of antibiotic consumption reported by the Swiss national surveillance [15] and western European countries such as Germany, France, the United Kingdom and Belgium [29,30]. In particular, data from the FOPH show a small reduction in antibiotic consumption in outpatient care with a most dramatic fall between 2015 and 2020. Similarly, in our study the decline in antibiotic prescriptions started in 2013, and also appears to coincide with the introduction of the Swiss strategies Smarter Medicine (Choosing Wisely Switzerland) and the Strategy on Antibiotic Resistance (StAR), which were implemented between 2014 and 2015 to optimise antibiotic consumption at national level [31,32]. Although no national data on antibiotic consumption were available for the period 2008–2013, the increase in antibiotic prescribing observed in our study is parallel to that observed for the same period in some European countries from the ESAC project [8].

Our study indicates that RTIs, UTIs, and skin infections are the most common indications for antibiotic prescribing in Swiss primary care. This finding is consistent with previous Swiss and international studies evaluating the consumption of antibiotics at the primary care level [15,33,34,35]. Although we cannot draw direct conclusions on the appropriateness of antibiotic prescribing using our dataset, it is expected that both RTIs and UTIs are, at least to a certain degree, inappropriately treated with antibiotics [21,22]. Especially, RTIs account for nearly half of all antibiotic prescriptions in primary care, and up to 64% of these are unnecessary or inappropriate both in the Swiss (41%) and other settings [6,22,36,37,38,39]. In our study, ciprofloxacin and norfloxacin accounted for 85% of all fluoroquinolone prescriptions. One of the two fluoroquinolones were prescribed to a third of all patients with a UTI diagnosis code. These antibiotics are often inappropriately prescribed for UTIs [21]. Thus, although the overall prescription rates of fluoroquinolones are declining, our study suggests that a relevant proportion of these prescriptions may still be inappropriate.

Our findings by seasonal fluctuation are similar to those both from Swiss [24] and international [40,41,42,43] studies showing that antibiotic prescribing is higher in winter than in summer months. Antibiotic consumption during the autumn and winter seasons in the continents of the Northern hemisphere has been demonstrated [44]. In particular, countries with high yearly use of antibiotics show heightened peaks of antibiotic prescribing in winter [5].

### 3.1. Factors Associated with Antibiotic Prescribing

Research shows that a minority of physicians prescribe the majority of antibiotics in the outpatient care setting [45]. Our results confirm this finding showing that the upper quarter of prescribers in our database prescribed almost 43% of all antibiotics. Results from our regression analysis suggest that more antibiotic prescribing is associated with increased consultation rates, and less prescribing is associated with lower rates of employment. These factors, relating to the amount of workload, are in line with research showing that high practice volume may result in higher (frequent and inappropriate) antibiotic prescribing [46,47]. In our study, RTI and UTIs were the clinical indications most commonly prescribed with antibiotics, which is in line with research showing that GPs with a high practice volume are more likely to (inappropriately) prescribe antibiotics for RTIs overall [46,47] and viral RTIs [48]. Research has also shown that GPs tend to prescribe more when they feel they have less available time per patient [47]. Studies from Europe [47] and Canada [48] show that GPs with longer ‘years (of experience) in practice’ are more likely to prescribe unnecessary (frequently and inappropriately) antibiotics. In our univariable analysis, however, this factor was not a significant predictor of antibiotic prescribing, which agrees with other Swiss [21,23,49] studies in the GP setting. A sensitivity analysis (results not shown) confirmed that, after correcting for confounding, the rates of antibiotic prescribing were not significantly different by GPs with “no” self-dispensing than by GPs with self-dispensing (*p* = 0.145).

Identification of the patient characteristics that are associated with increased (inappropriate) antibiotic prescriptions is essential too. In our study, patients’ increasing age and being a woman increased the likelihood of receiving an antibiotic prescription. In our sensitivity analysis, after excluding patients with UTIs, however, gender was no longer associated with antibiotic prescribing (*p* = 0.505). These findings are in line with previous studies from Europe [50,51], the USA [52], and Australia [53].

### 3.2. Strengths and Limitations of the Study

Our study is the first study in the field assessing the patterns and associated factors of antibiotic prescribing exclusively in the GP setting. Our analysis includes all data available on prescribing of systemic antibiotics regardless of the clinical indication, or whether GPs were low or high prescribers, in contrast with studies assessing the prevalence, predictors, or quality of antibiotic prescribing [21,22,23,24,25]. Moreover, our analysis based on aggregated data over 12 years shows trends of antibiotic prescribing that are similar to those reported by individual prospective studies and the national surveillance, thus supporting the validity of our results.

Our study was limited by a sample that is predominantly representative of the German-speaking area of Switzerland. Compared to sales or health insurance claims data, we could not calculate the number of “Defined Daily Doses” (DDDs) per 1000 inhabitants per day as this would depend on patient data that were not uniformly available in our database. Our analysis thus could not provide a more in-depth comparison for the national surveillance data. Finally, the number of GPs collaborating in the FIRE database increased over the last decade, changing the sample size of our study over the observation period. The confidence intervals across the GPs’ sample sizes over time were similar and non-significant, however.

### 3.3. Implications for Future Research, Policy and Practice

Several initiatives, both Swiss [12,31,32] and international [8,13,54] have responded to the emergency call from the WHO [10] to contain ABR by establishing surveillance on antibiotic consumption as a central element. As such, tackling inappropriate antibiotic use aims at both quantitative and qualitative aspects [55]. In our study, prescriptions of fluoroquinolones, macrolide and lincosamide declined, although at the expense of their potentially inappropriate use and increased use of BSP. Future health policymaking should consider this quantitative-qualitative phenomenon.

At the national level, several aspects of antibiotic stewardship could be considered to improve antibiotic prescribing in primary care. Continued efforts could help to sustain and further reduce the use of antibiotics in the AWaRe Watch group, fluoroquinolones, macrolide and lincosamides in particular. In addition, specific interventions aiming to halt and reduce the increased use of BSP could be implemented. Using aminopenicillins without BLI is appropriate for many upper RTIs [56,57], for example, but in our study, a combination of penicillin and BLI was prescribed 4.5 more often. The interventions could also target the inequality in antibiotic prescriptions between GPs. Proper identification of “high prescribers” could be crucial to improve antibiotic prescribing [58]. Especially in settings with limited resources, focusing on the group of “high-prescribers” could maximise the impact of future interventions.

Another key factor that future antibiotic stewardship interventions should consider is how gender influences prescribing. Whether this fact is only affected by the tendency in women to seek primary care more often than men [59] needs to be evaluated in further studies. In our study, excluding the patients with UTIs from our analyses showed gender as a non-significant predictor of antibiotic prescribing, which demonstrates that UTIs can be a significant confounding variable of antibiotic prescribing in women in a sample assessing overall antibiotic prescribing.

## 4. Materials and Methods

### 4.1. Study Design and Setting

We conducted a retrospective cross-sectional analysis using data from health EMRs contained in the database of Family Medicine ICPC (International Classification of Primary Care)-Research using EMR (FIRE) [26]. FIRE was established in 2008 and collects anonymised routine patient medical and health data provided by a network of General practitioners (GPs) from the German-speaking area of Switzerland. By April 2020, the database had accumulated data from approximately 550 GPs representing 10.5% of all registered GPs working in the German-speaking area and comprising more than 780,000 patients with more than 9.4 million consultations.

### 4.2. Participants’ Inclusion and Exclusion Criteria and Operational Definitions

We included all FIRE data available from 2008 to April 2020 from patients within the age of ≥18 and ≤100 years and who were prescribed with at least one antibiotic for systemic use. We identified all antibiotic prescriptions within FIRE based on 1) the WHO Anatomical Therapeutic Chemical Classification System (ATC) codes [60] and 2) Swiss pharma codes, which are unique numbers for the identification of a drug and dosage. We selected all systemic antibacterial drugs, antimycobacterials, and drugs with ATC codes specific for intestinal antiinfectives (see Supplementary Materials). We excluded ATC codes of topical antibacterial drugs, antimycotics, and antivirals; and data for which the date of first antibiotic prescription, for a single patient, coincided with the date of first consultations. Research using the FIRE database does not require ethical approval since it does not fall under the scope of the Federal Act on Research involving human beings (Human Research Act; Local Ethics Committee of the Canton of Zurich, BASEC-Number: Req-2017-00797).

### 4.3. Classification of Antibiotics and Diagnoses

We grouped all antibiotics according to drug classes and used WHO AWaRe to classify all selected antibiotics [28]. The WHO AWaRe classification consists of three stewardship groups that emphasise the importance of their optimal use and their potential for antibiotic resistance: Access, Watch, and Reserve. Diagnosis codes based on the current ICPC-2 code system [61] were available for a subgroup of patients, and we related these diagnoses to ATC codes.

### 4.4. Data Variables and Analysis

We characterised antibiotic prescriptions by patients and GPs’ characteristics (age, gender), canton of GPs’ practice, and rates and type of antibiotics by ATC codes and AWaRe class. We calculated the number of patients or events with their corresponding proportions (n and %) to describe categorical data, and the mean, standard deviations (SD) and ranges [min-max] to describe numerical data. We performed subgroup analyses of patient records with available ICPC-2 diagnosis (general and infectious diseases) codes to identify the most common clinical indications for antibiotic prescribing. These analyses used data from two weeks before and two weeks after the first antibiotic prescription to make sure of a connection between the diagnoses and antibiotic prescriptions. We used the Lorenz curve [62] to analyse and describe the (un-)equality in distribution of antibiotic prescriptions among GPs. The Gini coefficient represents the area under the Lorenz curve. It is a measure of deviation from equal distribution. A Gini coefficient ranges from “0” to “1”, which implies perfect equality or total inequality, respectively. The further the Lorenz curve deviates from perfect equality, the higher the Gini coefficient and the higher the inequality. A recommended interpretation for Gini values includes low inequality for a coefficient of 0.20 and extreme inequality for a coefficient of 0.50 [63].

We performed univariable and multivariable analyses to assess the association between patients’ and GPs’ characteristics and antibiotic prescribing. We included patients’ and GPs’ characteristics as predictors or fixed effects including age, gender, number of consultations per year, years in practice, employment level and status, and practice type. We performed linear random-effects mixed model regression analysis at the GP cluster-level. We corrected the model for GP consultations using a dichotomous variable (< or ≥6000 GP consultations per year). In the multivariable model analysis, we considered relevant effects together by constructing a model using variables with potential relevance for subgroup analyses, and which had a significance level of *p* ≤ 0.2 in the univariable analysis. Using stepwise backward elimination, we developed the final multivariable model with the best fit for the outcome of interest. We performed sensitivity analyses to identify potential confounders of antibiotic prescribing using univariable analyses based on the number of prescriptions for the sample of patients with available ICPC codes. We also considered the type of dispensing license in a sensitivity analysis. We present the results as effect estimates with their corresponding 95% confidence intervals (CI) and intraclass correlation coefficient and report the summary statistics.

We performed all analyses using the statistical software package R (version 3.6.1) and considered *p* < 0.05 statistically significant for all tests [64]. For all analyses with specific GPs’ characteristics (e.g., age, gender, years in practice, consultations, type of practice), we used a validated dataset from 2018 [65] since differentiation between single and group GP practices is not yet possible in our database.

## 5. Conclusions

In Swiss (German-speaking) general practice, RTIs, UTIs and skin infections are the most common clinical indications prescribed with antibiotics. Amoxicillin plus BLI, ciprofloxacin and clarithromycin are the most commonly prescribed antibiotics. In keeping with national and European surveillance, we found a decline in prescriptions of fluoroquinolones, macrolides and lincosamides, however, at the expense of their potentially inappropriate use and increased use of BSP. Several patients and GPs’ characteristics were associated with antibiotic prescribing. Health policy initiatives engaged in antibiotic stewardship could use these findings to inform future interventions tailored to the factors associated with antibiotic prescribing.

## Figures and Tables

**Table 1:** Distribution of 206,599 antibiotic prescriptions in 112,378 patients in Swiss primary care for the period 2008–2020.

Class Number, n	Antibiotic Class	Prescriptions, n (%)
1	Broad spectrum penicillins	75,534 (36.5)
2	Fluoroquinolones	33,900 (16.4)
3	Macrolides, Lincosamides	28,589 (13.8)
4	Other antibacterials ^1^	23,268 (11.3)
5	Sulfonamides and Trimethoprim	18,380 (8.9)
6	Tetracyclines	9670 (4.7)
7	Cephalosporins	7886 (3.8)
8	Intestinal antiinfectives ^2^	5364 (2.6)
9	Narrow spectrum penicillins	3160 (1.5)
10	Antimycobacterials	732 (0.4)
11	Carbapenems	53 (0.0)
12	Glycopeptides	45 (0.0)
13	Aminoglycosides	17 (0.0)
14	Monobactams	1 (0.0)

^1^ This group consists of drugs in the ATC group J01XB-J01XX; ^2^ This group consists of drugs in the ATC groups A07AA and P01AB01. ATC, WHO Anatomical Therapeutic Chemical Classification System codes; n, number.

**Table 2:** Top 15 clinical indications for antibiotic prescribing in Swiss primary care.

Clinical Indications	ICPC-2 Code ^1^	Prescriptions, n
Respiratory tract		11,039
Upper respiratory tract infections	R74	2516
Pneumonia	R81	1777
Acute/chronic sinusitis	R75	1664
Cough	R05	1404
Acute Tonsillitis	R76	1271
Acute Bronchitis / Bronchiolitis	R78	973
Throat symptoms	R21	720
Streptococci pharyngitis	R72	714
Urinary tract infection	U71	5898
Skin infection	S76	1000
Diverticulosis / Diverticulitis	D92	971
Abdominal Pain	D06	599
Fever	A03	571
Insect bite	S12	550
Acute otitis media	H71	549

^1^ ICPC-2, International Classification of Primary care 2 code system; n, number.

**Table 3:** Antibiotic prescriptions for infectious diseases by ICPC-2 diagnosis and anatomic groups.

Infectious Diseases	ICPC Category ^1^	Overall Prescriptions, n	Overall Prescriptions, %
		21,021	100
Respiratory	R71-83	9968	47.4
Urological	U70-72	6514	31.0
Skin	S03,09-11,70-76, 84, 95	1409	6.7
Ear	H70-74	926	4.4
Digestive system	D70-73	847	4.0
Male genital	Y70-76	503	2.4
General and unspecified	A70-78	467	2.2
Eye	F70-73	169	0.8
Musculoskeletal	L70	78	0.4
Female genital	X70-74, 90-92	80	0.4
Blood, Blood Forming Organs and Immune Mechanism	B70-71	29	0.1
Cardiovascular	K70-71	16	0.1
Neurological	N70-73	11	0.1
Endocrine/Metabolic and Nutritional	T70	4	0.0

^1^ ICPC-2, International Classification of Primary Care 2 code system; n, number.

**Table 4:** Mixed model regression analysis on antibacterial prescriptions in 2018.

Predictor (Reference, Where Applicable)	Univariable Analysis			Multivariable Analysis n = 26,589, GPs = 240	
	Prescriptions and GPs, n	Estimates (95% CI)	*p*	Estimates (95% CI)	*p*
GP Characteristics				ICC = 0.09	
Age	n = 26,595, GP = 240	0.005 (−0.001, 0.011)	0.080		
N. consultations ≥ 6000 (<6000)	n = 26,595, GP = 240	0.355 (0.252, 0.457)	<0.001	0.281 (0.163, 0.398)	<0.001
Female gender (male)	n = 26,595, GP = 240	−0.205 (−0.316, −0.094)	<0.001	−0.079 (−0.199, 0.042)	0.204
Years in practice	n = 22,808, GP = 204	0.006 (−0.0004, 0.012 )	0.068		
Self-Employed (employee)	n = 24,604, GP = 216	0.089 (−0.035, 0.213)	0.161		
Employment level (100%)	n = 26,595, GP = 240				
<50%		−0.483 (−0.694, −0.266)	<0.001	−0.279 (−0.507, −0.051)	0.017
50–79%		−0.271 (−0.400, −0.143)	<0.001	−0.100 (−0.247, 0.047)	0.183
80–99%		−0.096 (−0.235, 0.044)	0.179	−0.058 (−0.193, 0.077)	0.402
Practice Characteristics					
Type of practice (single practice)	n = 26,595, GP = 240				
Double practice		−0.136 (−0.451, 0.179)	0.399		
Group practice		−0.086 (−0.247, 0.075)	0.297		
Patient Characteristics					
Age	n = 26,595, GP = 240	0.002 (0.001, 0.003)	<0.001	0.002 (0.001, 0.003)	<0.001
Male gender (female) *	n = 26,589, GP = 240	−0.231 (−0.270, −0.193)	<0.001	−0.234 (−0.272, −0.196)	<0.001

Note. Linear model of number of prescriptions with patient and general practitioners (GPs) characteristics as fixed effects and GPs as random effects (mixed model)**.** n, number; CI, Confidence interval; ICC, Intraclass correlation. *: Sensitivity analyses: after excluding patients with ICPC (International Classification of Primary Care) codes available for urinary tract infections, gender was no longer associated with antibiotic prescribing (*p* = 0.505).

## Supplementary Materials

Additional supporting material is contained in the Supplementary Materials and it is available online at https://www.mdpi.com/2079-6382/9/11/837/s1. Table S1: Full list of all antibiotic prescriptions in Swiss primary care; Table S2: Gender distribution for quinolone prescriptions.
