# Supplementary material for: Time Trends and Factors Associated with Antibiotic Prescribing in Swiss Primary Care (2008 to 2020)
Source: Antibiotics (Basel). 2020 Nov 23;9(11):837. doi: 10.3390/antibiotics9110837 (PMC7700253; doi:10.3390/antibiotics9110837)
Supplement: Supplementary file 1 [file antibiotics-09-00837-s001.pdf]

Time trends and factors associated with antibiotic prescribing in Swiss primary care (2008 to 2020)

Table S1: Full list of all antibiotic prescriptions in Swiss primary care.

Antibiotics	ATC code	Prescriptions, n (%)
		**N=206,599**
**Tetracyclines**		**9670 (4.7)**
Doxycycline	J01AA02	8316 (4.0)
Lymecycline	J01AA04	395 (0.2)
Minocycline	J01AA08	959 (0.5)
**Narrow spectrum penicillins**		**3160 (1.5)**
Benzylpencillin	J01CE01	100 (0.0)
Phenoxymethylpenicillin	J01CE02	2793 (1.3)
Benzathine phenoxymethylpenicillin	J01CE10	24 (0.0)
Flucloxacillin	J01CF05	243 (0.1)
**Broad spectrum penicillins**		**75,534 (36.5)**
Amoxicillin	J01CA04	13,794 (6.7)
Amoxicillin / betalactamase inhibitor	J01CR02	61,733 (29.8)
Piperacillin / BLI	J01CR05	7 (0.0)
**Cephalosporins**		**7886 (3.8)**
Cefazolin	J01DB04	5 (0.0)
Cefuroxime	J01DC02	6181 (3.0)
Cefamandole	J01DC03	1 (0.0)
Cefaclor	J01DC04	43 (0.0)
Cefprozil	J01DC10	185 (0.1)
Ceftriaxone	J01DD04	644 (0.3)
Cefixime	J01DD08	86 (0.0)
Cefpodoxime	J01DD13	695 (0.3)
Ceftibuten	J01DD14	40 (0.0)
Cefepime	J01DE01	5 (0.0)
Ceftolozane / BLI	J01DI54	1 (0.0)
**Monobactams**		**1 (0.0)**
Aztreonam	J01DF01	1 (0.0)
**Carbapenems**		**53 (0.0)**
Meropenem	J01DH02	1 (0.0)
Ertapenem	J01DH03	49 (0.0)
Imipenem and cilastatin	J01DH51	3 (0.0)
**Sulfonamides and Trimethoprim**		**18,380 (8.9)**
Sulfadiazine	J01EC02	2 (0.0)
sulfamethoxazole and trimethoprim	J01EE01	18,378 (8.9)
**Macrolides, Lincosamides**		**28,589 (13.8)**
Erythromycin	J01FA01	87 (0.0)
Spiramycin	J01FA02	2 (0.0)
Roxithromycin	J01FA06	4 (0.0)
**Clarithromycin**	**J01FA09**	**14,622 (7.1)**
Azithromycin	J01FA10	10,461 (5.0)
Clindamycin	J01FF01	3413 (1.6)
**Aminoglycosides**		**17 (0.0)**
Tobramycin	J01GB01	14 (0.0)

Antibiotics	ATC code	Prescriptions, n (%)
Amikacin	J01GB06	3 (0.0)
**Fluoroquinolones**		**33,900 (16.4)**
Ofloxacin	J01MA01	171 (0.1)
Ciprofloxacin	J01MA02	21,770 (10.5)
Norfloxacin	J01MA06	6985 (3.4)
Levofloxacin	J01MA12	1430 (0.7)
Moxifloxacin	J01MA14	3544 (1.7)
**Glykopeptides**		**45 (0.0)**
Vancomycin	J01XA01	40 (0.0)
Teicoplanin	J01XA02	5 (0.0)
**Other** [1]		**23,268 (11.2)**
Colistin	J01XB01	17 (0.0)
Fusidic acid	J01XC01	334 (0.2)
Metronidazole	J01XD01	3 (0.0)
Nitrofurantoin	J01XE01	5633 (2.7)
Fosfomycin	J01XX01	17,252 (8.3)
Linezolid	J01XX08	15 (0.0)
Daptomycin	J01XX09	14 (0.0)
**Antimycobacterials**		**732 (0.4)**
Rifampicin	J04AB02	417 (0.2)
Rifabutin	J04AB04	21 (0.0)
Isoniazid	J04AC01	127 (0.1)
Ethambutol	J04AK02	54 (0.0)
Rifampicin and isoniazid	J04AM02	74 (0.0)
Rifampicin, pyrazinamide and isoniazid	J04AM05	20 (0.0)
Rifampicin, pyrazinamide, ethambutol and isoniazid	J04AM06	19 (0.0)
**Intestinal antiinfectives** [2]		**5364 (2.6)**
Metronidazole	P01AB01	5209 (2.5)
Vancomycin oral	A07AA09	60 (0.0)
Paromomycin	A07AA06	23 (0.0)
Rifaximin	A07AA11	67 (0.0)
Fidaxomicin	A07AA12	5(0.0)

Note. BLI, Beta-Lactamase Inhibitors; ATC, WHO Anatomical Therapeutic Chemical Classification System; n, number.

[1] This group consists of antibiotics in the ATC group J01XB-J01XX.

[2] This group consists of all antibiotics in the ATC group A07AA (Intestinal antiinfectives – antibiotics), and metronidazole (P01AB01).

Table S2: Gender distribution for quinolone prescriptions.

		Overall, n (%)	Female, n (%)	Male, n (%)	p
**Quinolones**		**N=33,900**	**20,384**	**13,512**	**<0.001**
Age (mean, SD)		59.16 (19.95)	58.54 (21.07)	60.09 (18.09)	**<0.001**
Ofloxacin	J01MA01	171 (0.5)	81 (0.4)	90 (0.7)	**0.001**
Age (mean, SD)		58.02 (17.26)	60.48 (18.14)	55.80 (16.22)	0.077
Ciprofloxacin	J01MA02	21,770 (64.2)	11,287 (55.4)	10,481 (77.6)	**<0.001**
Age (mean, SD)		58.76 (19.55)	57.77 (20.87)	59.84 (17.95)	**<0.001**
Norfloxacin	J01MA06	6,985 (20.6)	6,262 (30.7)	723 (5.4)	**<0.001**
Age (mean, SD)		60.69 (21.88)	59.79 (22.15)	68.50 (17.56)	**<0.001**
Levofloxacin	J01MA12	1,430 (4.2)	783 (3.8)	647 (4.8)	**<0.001**
Age (mean, SD)		59.58 (18.78)	59.89 (19.18)	59.21 (18.30)	0.492
Moxifloxacin	J01MA14	3,544 (10.5)	1,971 (9.7)	1,571 (11.6)	**<0.001**
Age (mean, SD)		58.44 (18.85)	58.37 (19.26)	58.54 (18.31)	0.785

Notes. SD, Standard Deviation; n, number; p, p-value.
